# Pentacene derivative/DTTCNQ cocrystals: alkyl-confined mixed heterojunctions with molecular alignment and transport property tuning†
†Electronic supplementary information (ESI) available: Experimental procedures, the preparation of the TMTES-P/DTTCNQ single crystal, the intermolecular distances in the cocrystals, short-contact interactions, infrared absorption spectra, solid-state UV-vis-NIR absorption spectra, and ESR, AFM, TEM and crystal data. CCDC 1918869 (cocrystal P1); and 1918870 (cocrystal P2). For ESI and crystallographic data in CIF or other electronic format see DOI: 10.1039/c9sc04807c


**DOI:** 10.1039/c9sc04807c

**Published:** 2019-10-14

**Authors:** Yudong Ma, Yecheng Zhou, Jianqun Jin, Wei Wang, Xitong Liu, Haixiao Xu, Jing Zhang, Wei Huang

**Affiliations:** a Key Laboratory for Organic Electronics and Information Displays & Jiangsu Key Laboratory for Biosensors , Institute of Advanced Materials (IAM) , Jiangsu National Synergetic Innovation Center for Advanced Materials , Nanjing University of Posts & Telecommunications , 9 Wenyuan Road , Nanjing 210023 , China . Email: iamjingzhang@njupt.edu.cn ; Email: wei-huang@njtech.edu.cn; b Department of Physics , Southern University of Science and Technology , Shenzhen 518055 , China; c Shaanxi Institute of Flexible Electronics (SIFE) , Northwestern Polytechnical University (NPU) , 127 West Youyi Road , Xi'an 710072 , Shaanxi , China

## Abstract

Organic cocrystals are formed *via* the self-assembly of donor and acceptor constituents, which are mixed together through weak noncovalent interactions. Although they reveal unique physical features, their synthesis still faces major drawbacks for the introduction of more potential semiconductors. Herein, we first report soluble pentacene derivative (TMTES-P) based complexes, with suitable alkyl terminal groups, enabling the location of 4,8-bis(dicyanomethylene)-4,8-dihydrobenzo[1,2-*b*:4,5-*b*′]-dithiophene (DTTCNQ) in the crystal lattice, thereby allowing the cocrystallization of a binary system on demand. To our surprise, *via* varying growth conditions, molecular disorders could be removed due to existing short-contacts as the locking force, and even the carrier charge could be changed. This organic donor–acceptor system presents unconventional insights: charge polarity control over (opto)electronic devices with a supramolecular driving force as the directional alignment guide.

## Abstract 2

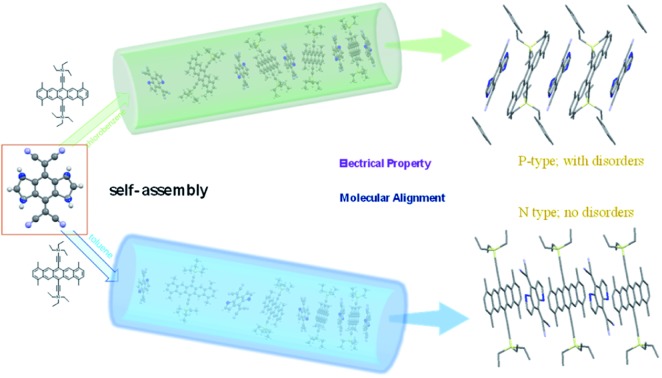
Soluble pentacene-based complexes were successfully prepared and short contact interactions induced alignment driving forces to eliminate C/S disorders. Cocrystal packing and charge transport properties were tailored by adjusting the solvent.

## Introduction

In addition to long-term conductivity and superconductivity properties research, organic charge-transfer complexes have now been reported in many unpredicted functional applications, such as evolutional semiconducting charge-transport,^1^ light emitting,^2^ nonlinear optics,^3^ photovoltaics properties,^4^ ferroelectrics,^5^ stimuli-responsibility^6^ and pharmaceutics.^7^ In recent years, as a novel semiconductor class, a series of organic bi-component complexes gave rise to multiple polarity charge transport features by incorporating fine-tuned donors and acceptors.^8^ Computational methods have also been developed for a newly-emerging interdigitated supramolecular model to reveal structure–property relationships considering molecular-level heterojunction interactions. For example, an energy-splitting method accounting for frontier orbitals of an A–D–A/D–A–D triad^9^ and a partition method involving of other bridge orbitals,^10^ in turn provide helpful insight for a rational design of new organic binary materials.^11^ However, Most electron-rich donors contain short π-conjugated cores and no/short alkyl substitutions in these mixed network systems; for example, perylene,^12^ tetracene^13^ or coronene,^14^ are accompanied by the solubility, symmetry and stoichiometry problems which led to the limitation of produce approach, purity and yield issues. Intriguingly, soluble dialkylated benzothienobenzothiophenes can form isomorphous complexes with fluorinated derivatives of tetracyanoquinodimethane where substituted alkyl chain lengths affected interplanar π-stack angles because of steric hindrance.^15^ So far, no linear five benzene ring compounds, known as the popular semiconductor pentacene and its derivatives, have been successfully applied in constructing highly ordered donor–acceptor cocrystals, except organic solid solutions of isometric silylethynylated tetraazapentacene and silylethynylated pentacene,^16^ and a 2 : 1 cocrystal of nonaromatic 6,13-dihydropentacene and pentacene.^17^ Hence, the introduction of pentacene, and especially its soluble derivatives^18^ widely investigated in electronic and theoretical researches,^19^ would ensure the related semiconducting cocrystallization system as such is both challenging and promising.

**Fig. 1:**
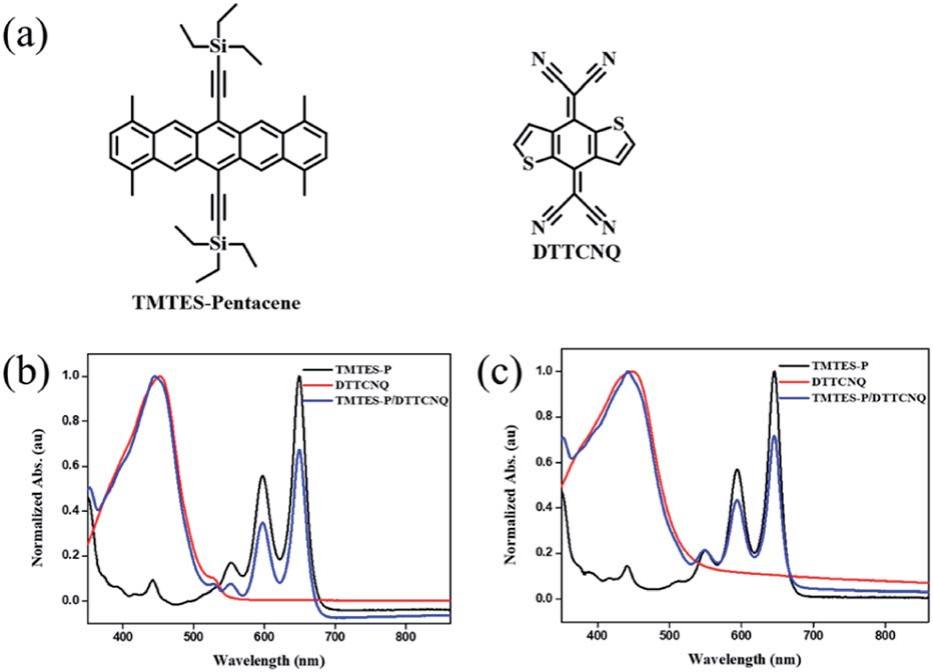
(a) Chemical structures of the donor TMTES-P and the acceptor DTTCNQ. UV-vis absorption spectra of dilute (b) chlorobenzene and (c) toluene solutions of TMTES-P, DTTCNQ and a 1 : 1 mix.

The organic complexes are defined as supramolecular frameworks built under various weak intermolecular interactions. Therefore, during the assembling process to the solid-state superstructure, two distinct original organic compounds reorganize into one crystalline lattice driven by directional charge transfer (CT), hydrogen-bonding and other non-bonding interactions,^20^ which are crucial for control of the organic heterojunction system, and thus the design of a novel binary material that can take advantage of them for a revolutionary supramolecular chemistry investigation.^21^ For example, 4,8-bis(dicyanomethylene)-4,8-dihydrobenzo[1,2-*b*:4,5-*b*′]-dithiophene (DTTCNQ) (Fig. 1a), is a thiophene-fused π-extended conjugated TCNQ derivative which displayed electron mobility of 0.03 cm^2^ V^–1^ s^–1^ in thin films^22^ and constructed highly conducting and semiconducting charge-transfer complexes with common donors.^23^ But both the single crystal and cocrystal structures suffer DTTCNQ orientation disorders with a pseudo symmetry of mm,^24^ while in our minds cocrystal engineering strategy is a good way to control disorders by using weak short-contact interaction guidance.^25^ Introducing a new functional donor material into a highly ordered binary cocrystal platform is of great importance for organic semiconductor class developments, a deep understanding of structure–property relationships and supramolecular chemistry.

## Results and discussion

Starting from the parent compound, 1,4,8,11-tetramethyl-6,13-triethylsilylethynyl pentacene (TMTES-P, Fig. 1a) as a typical p type semiconductor with a thin film based device, mobility up to 2.5 cm^2^ V^–1^ s^–1^ was selected as the electron donor with a less crowded ethyl end compared to classical TIPS-P and additional methyl groups attached to pentacene skeleton.^26^ TMTES-P has a planar π-core and good crystallinity, which could act as a good candidate to synthesize a binary complex with DTTCNQ. Fig. 1b and c show a normalized UV-vis absorption spectrum of TMTES-P, DTTCNQ and corresponding mixtures in chlorobenzene and toluene. For the 1 : 1 mixed chlorobenzene or toluene solution, multiple peaks of TMTES-P at 552 nm, 597 nm and 649 nm did not show a noticeable shift. However, a blue shift of 8 nm or 7 nm was found as a pure DTTCNQ absorption band at 449 nm or 452 nm, indicating intermolecular charge-transfer interactions of TMTES-P and DTTCNQ in the solution state and the possibility of complex formation.

**Fig. 2:**
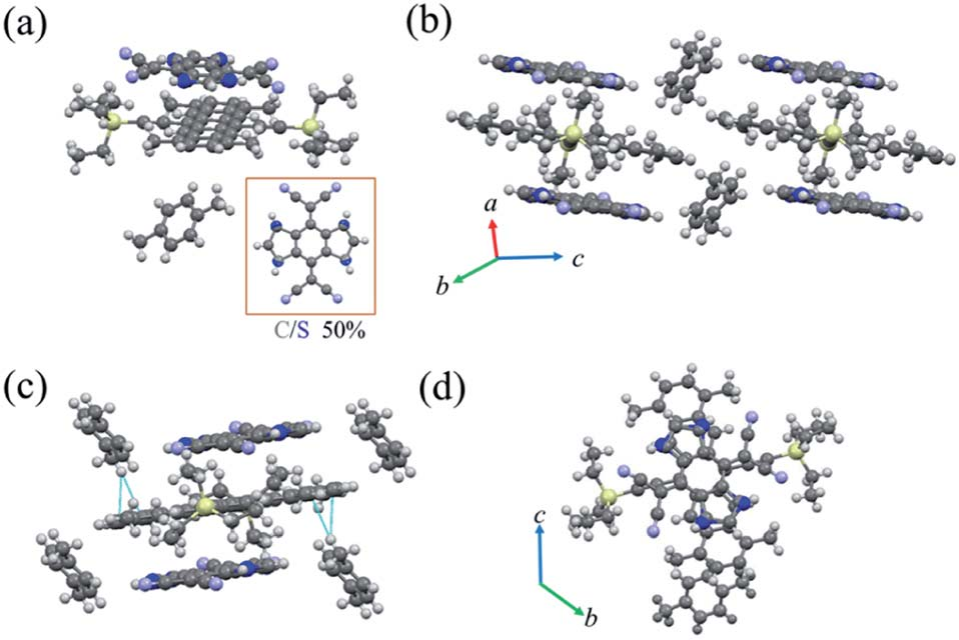
(a) The molecular structure of cocrystal P1 with carbon–sulfur atom disorders at the 1, 2, 4 and 5-positions of the quinoid ring. (b) The crystal stacking of the donor–acceptor system (π–π stacking pattern). (c) Short-contact interactions in the cocrystal P1. (d) The overlap pattern between TMTES-P and DTTCNQ (viewed along the *a*-axis).

Then, after solvent evaporation of the mixture solutions (chlorobenzene or toluene) in a Petri dish, a very simple and quick route (Fig. S1†), two different TMTES-P based cocrystals were prepared depending on the solvent. Both of the resulting cocrystals displayed dark colour and similar needle-like shapes (further detailed crystal structure information is listed in Table S1†). Obtained from the chlorobenzene solution, cocrystal P1 consisted of 1 : 1 : 1 TMTES-P, DTTCNQ and an inserted xylene molecule (Fig. 2a), crystallizing into the triclinic space group *P*1[combining macron] with unit cell parameters of *a* = 7.727(4) Å, *b* = 14.153(7) Å, *c* = 15.295(7) Å, *α* = 109.638(12)°, *β* = 102.453(14)° and *γ* = 103.402(13)°. The xylene molecule is hypothesized to be from impurity of the solvent. In DTTCNQ, the thiophene units were disordered with sulfur atoms appearing in the 1, 2, 4 and 5-positions of the quinoid ring with 50% occupancy, as with other reported DTTCNQ based complexes.^22^ For complex P1, the donor and acceptor molecules stack alternatively into mixed columns along the *a*-axis (Fig. 2b). A dihedral angle of 1.64° was observed between the π-core plane of TMTES-P and the neighbouring DTTCNQ in the same column, and two of the cyano groups deviated from the main-backbone plane of the DTTCNQ molecule by 0.441 Å. The intermolecular distance was around 3.59 Å as determined from the crystal structure (Fig. S2†), which suggested weak π–π interactions along the DA mixed packing direction. On the other hand, no non-bond short-contacts existed in either the stacking direction or adjacent columns as shown in Fig. 2c. Only a few existing non-bond interactions between the ethyl/terminal phenyl group and methyl of xylene were established to stabilize this supramolecular framework and confine the acceptor molecules (Fig. 2c and S3†). TMTES-P and DTTCNQ molecules shared the same symmetric center of the pentacene and quinoid rings along *a*-axis. The dicyanomethylene groups were close to the bulk alkyl ends, while the dithiophene fused benzene moiety overlapped almost completely with the pentacene backbone, with an offset of 1.40 Å to reduce the steric effect (Fig. 2d).

**Fig. 3:**
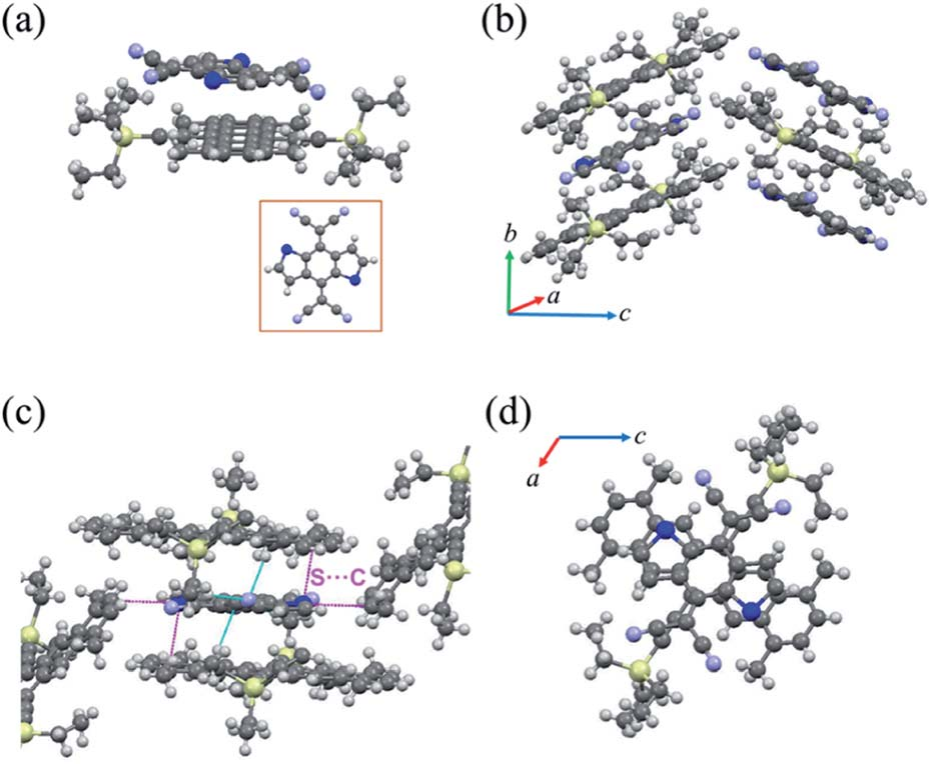
(a) The molecular structure of cocrystal P2. (b) The crystal stacking of cocrystal P2 (herringbone stacking pattern). (c) Short-contact interactions between the TMTES-P and DTTCNQ binary system. (d) The overlap pattern between TMTES-P and DTTCNQ (viewed along the *b*-axis).

When the solvent was changed to toluene, we obtained a new complex named cocrystal P2 *via* the exact same synthesis route. Complex P2 possessed a monoclinic symmetry with a unit cell in space group *P*2_1_/*n* and unit cell parameters *a* = 15.420(2) Å, *b* = 7.7100(11) Å, *c* = 21.640(3) Å, *α* = 90°, *β* = 104.049(4)°, *γ* = 90°. To our surprise, the xylene molecule was absent in this 1 : 1 TMTES-P/DTTCNQ cocrystal framework and so were the carbon-sulfur disorders (Fig. 3a). The TMTES-P and DTTCNQ molecules aligned into a strict mixed stack mode along the *b*-axis with a closer π–π distance of 3.52 Å, a tilt angle of 48.1° (Fig. 3b and S4†) and thus a larger packing density of 1.234 g cm^–3^ than that of cocrystal P1 (1.182 g cm^–3^). Along the π–π stacking direction, the angle between adjacent molecule planes was about 2.45°, and in order to facilitate the intermolecular interactions, two cyano groups bent up and down to the opposite sides by ∼0.578 Å away from the main backbone. There were N···H–C (2.697 Å) hydrogen bonds from the two bent cyano groups of DTTCNQ and the adjacent ethyl units of TMTES-P molecules to define the acceptors, suggesting the necessity of appropriate alkyl ends. Except for the stacking direction, S···C short contacts (∼3.496 Å) could also be observed between donor and acceptor molecules in adjacent columns (Fig. 3c and S5†), which were believed to locate the DTTCNQ strongly and thus cause the disorder elimination. Weak connects between one acceptor with the neighbouring four donor molecules constructed this specific network; ethyl groups with a relatively low steric hindrance contributed to fix the acceptors in the mixed stack and the short contacts between nearby columns created the atom orientation drive (Fig. 3c). The p–n junctions adopted the same overlap pattern as cocrystal P1 illustrated in Fig. 3d, with the centroidoffset of ∼1.57 Å.

The infrared (IR) spectra of both the complexes are shown in Fig. S6†. For pristine DTTCNQ, cocrystals P1 and P2, the C≡N stretching was observed at 2215, 2212, and 2211 cm^–1^ and the vibrational band shift can be assigned to intermolecular charge-transfer. In addition, the solid-state UV-vis-NIR absorption spectra of the two cocrystals was characterized (Fig. S7†), with a wide absorption range from ultraviolet to visible, even near infrared, which was also attributed to strong intermolecular charge-transfer (CT) interactions in the solid state. And band gaps of the P1 and P2 complexes were calculated to be about 0.8 eV and 0.78 eV based on a modified Kubelka–Munk equation. The cocrystal powders displayed a strong electron spin resonance (ESR) signal, and the calculated *g* factors were 2.0049 and 2.0050 for P1 and P2, revealed the existence of an unpaired electron (Fig S8†). The CT degrees were calculated to be 0.01 and 0.04 for cocrystals P1 and P2, respectively, based on Mulliken charge,^27^ which is lower than most other cocrystal systems.^28^ CT interactions between the DA pairs further ensured recombination of the fresh pentacene and TCNQ derivatives. In these two TMTES-P/DTTCNQ cocrystals, the proper triethylsilylethynyl units on both sides were supposed to build a particular platform for the formation of co-crystallization, and additional positioned short-contacts can avoid alignment disorders.

**Fig. 4:**
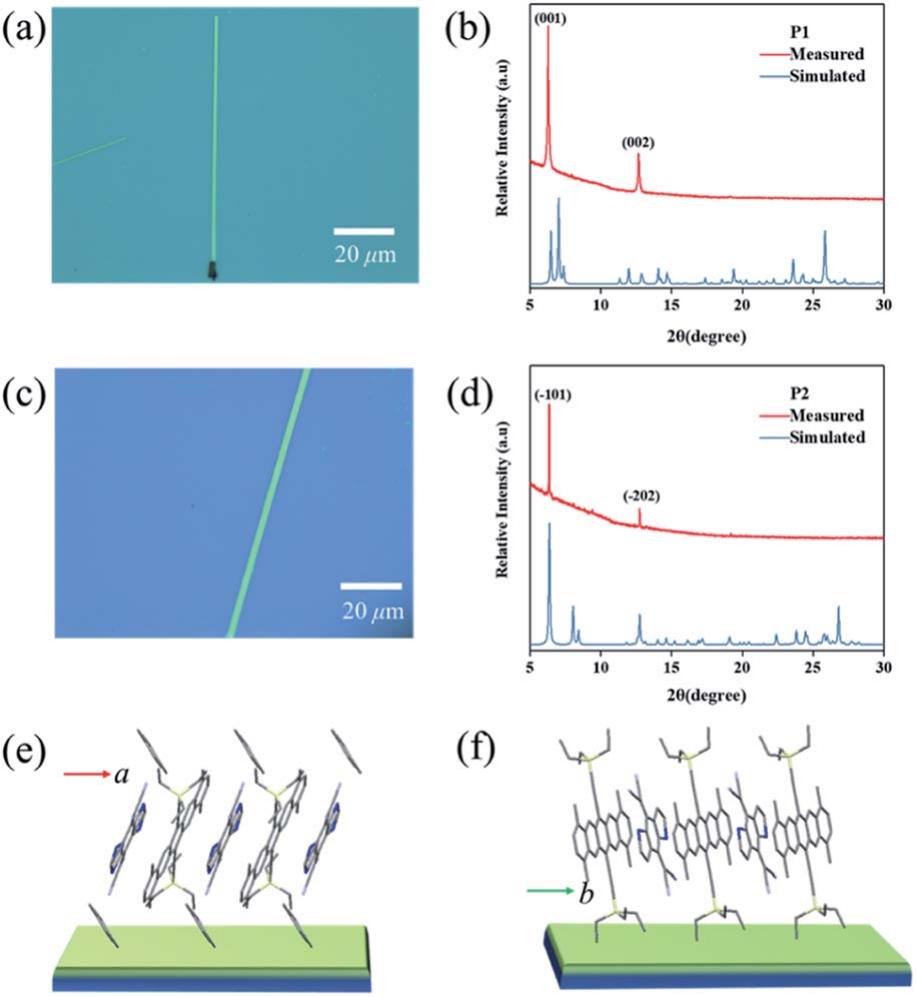
Optical micrographs of (a) P1 and (c) P2 micro/nanoribbons obtained *via* a solution drop-casting method. Measured and simulated X-ray diffraction (XRD) patterns of (b) P1 and (d) P2 microcrystals. In (b) and (d), the peaks are indexed with the lattice constants of their corresponding bulk crystals. The growth mode of the (e) P1 and (f) P2 micro/nanoribbons on the substrates.

Crystalline P1 and P2 micro/nanoribbons were prepared *in situ* by drop-casting their chlorobenzene and toluene solutions onto bare SiO_2_/Si or *n*-octadecyltrichlorosilane (OTS) modified SiO_2_/Si substrates (for details, see the experimental procedures†). Fig. 4a and c are optical micrographs of the as-prepared complexes P1 and P2 micro/nanoribbons, respectively, with a width from several hundreds of nanometers to several micrometers, and their lengths ranged from tens to even several hundred of micrometers. The thickness of the micro/nanoribbons was about 100 nm as detected by atomic force microscopy (Fig. S9†). The crystal structures of these microcrystals were further characterized by X-ray diffraction (XRD), which corresponded to their own bulk crystal data. For P1 micro/nanoribbons, the XRD patterns showed intense peaks at 6.24° and 12.64°, which were indexed as (001), and (002), respectively (Fig. 4b). The intense peak at 6.24° corresponds to a *d*-spacing of 1.40 nm, which is consistent with the *c*-axis, suggesting that the micro-cocrystals grew with a pentacene moiety standing on the substrates (Fig. 4e). In the case of P2 microribbons, the strong peaks at 6.24° and 12.64° were indexed as (–101) and (–202) (Fig. 4d), indicating DA alternative growth with a pentacene moiety lying on the substrates (Fig. 4f). Temperature dependent PXRD measurements (Fig. S10†) manifested good thermal stability of both cocrystals below 180 °C. And the growth direction of the cocrystals was supposed to be along the π–π interaction direction (*i.e.*, the mixed stacking direction). Transmission electron microscopy (TEM) and its corresponding selected area electron diffraction (SAED) images of P2 microribbons (Fig. S11†) proved that the [010] axis (mixed stacking direction) was parallel to the long direction of P2 crystals. These results revealed that not only the inner structure could be tailored, but also the growth mode differed greatly depending on the growth conditions (tuning the solvent).

**Fig. 5:**
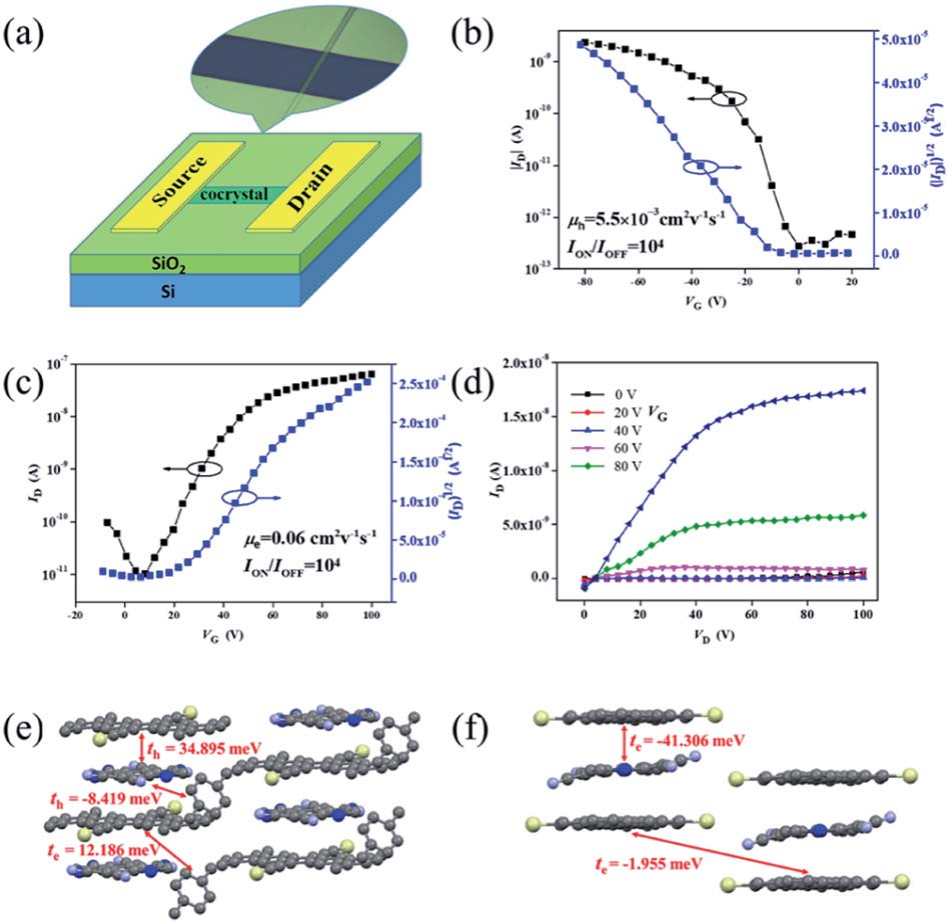
(a) A schematic diagram of the structure of the complex-based OFET and an optical image of an individual cocrystal device. (b) The transfer characteristics of the cocrystal P1 based device. (c) The transfer and (d) output curves of a cocrystal P2 based device. The main charge transport pathways and electronic coupling for holes and electrons in cocrystals (e) P1 and (f) P2. The ethyl groups have been omitted for clarity.

The charge transport properties of these cocrystals were then investigated by fabricating organic field-effect transistors (OFETs) of top-contact/bottom-gate configuration. Gold was thermally evaporated onto the microcrystals as source and drain electrodes through a copper grid as the shadow mask. Fig. 5a illustrates a schematic representation of one complex-based microcrystal transistor. All devices of two unique complexes were measured under air conditions and at room temperature. From the transfer characteristics (Fig. 5b), devices based on cocrystal P1 exhibited a hole-dominating transport feature with the highest mobility of 5.5 × 10^–3^ cm^2^ V^–1^ s^–1^. Transfer and output characteristics of transistors based on cocrystal P2 microribbons are presented in Fig. 5c and d. On the contrary, the non-xylene TMTES-P/DTTCNQ cocrystal P2 exhibited an n-type semiconducting behaviour, and the electron mobility reached to 0.06 cm^2^ V^–1^ s^–1^. This low charge-transfer degree was believed to favour a high on/off ratio of 10^4^ which is superior to most charge-transfer complexes. The charge transport property varied according to the intrinsic structure of the supramolecular system which led to the diverse π–π interaction, other short contacts and inserted additive.

To determine the charge polarity change of these two TMTES-P/DTTCNQ cocrystals, we evaluated their electronic couplings based on the crystal structure along possible transport pathways using the unperturbed density matrix of the dimer Fock operator.^29^ As presented in Fig. 5e, in cocrystal P1, the main transport pathway was found to be along the direction of π–π stacking (*a*-axis) and the hole transfer integral was 34.895 meV between adjacent donor and acceptor pairs. Insertion of a xylene molecule was supposed to hinder efficient hole transport and induce a relatively low hole mobility despite the high transfer integral.^14^ Although an electron transfer integral (12.186 meV) between a TMTES-P/xylene molecule existed, it did not match the crystal growth and measured direction required, thus, no effective electron channel appeared. In cocrystal P2 (Fig. 5f), the TMTES-P : DTTCNQ dimer along the π–π stacking direction revealed effective transfer integrals of –41.306 meV for the electrons, suggesting the n-type nature of cocrystal P2. This calculated result is consistent with our measurements.

## Conclusions

In summary, we successfully prepared a new family of supramolecular complexes consisting of a soluble pentacene derivative and DTTCNQ *via* an easy and feasible method. By just varying the solvent, two totally different cocrystals were produced. Compared with cocrystal P1, with the existence of xylene molecules to stabilize the binary framework, cocrystal P2 formed a more tightly slipping structure and additional directional short-contacts to eliminate DTTCNQ disorders. Furthermore, suitable ethyl groups were also proved to confine the acceptor, which was essential for the innovative architecture construction. The solvents and substituents had a great impact on this complicated recombination and even the standing pattern on the substrates. Electrical characterizations of both these complexes demonstrated that the cocrystals exhibited charge transport from p-type (P1) to n-type (P2) as tailored structures. Our work opens a new platform for the design, preparation and study of donor–acceptor complexes of high co-crystallinity. Importantly, we believe that this supramolecular chemistry tuning will be promising for molecular alignments, electrical properties, and, furthermore, even chiral selectivity.

## Conflicts of interest

There are no conflicts to declare.

## Supplementary Material

Supplementary information

Crystal structure data
