# Supplementary material for: Pentacene derivative/DTTCNQ cocrystals: alkyl-confined mixed heterojunctions with molecular alignment and transport property tuning
Source: Chem Sci. 2019 Oct 14;10(48):11125–9. doi: 10.1039/c9sc04807c (PMC7069240; doi:10.1039/c9sc04807c)
Supplement: Supplementary file 1 [file SC-010-C9SC04807C-s001.pdf]

Electronic Supplementary Material (ESI) for Chemical Science.
This journal is © The Royal Society of Chemistry 2019

Electronic Supporting Information

Pentacene derivative/DTTCNQ cocrystals: Alkyl-confined mixed heterojunctions with molecular alignment and transport property tuning

Yudong Ma,[a] Yecheng Zhou,[b] Jianqun Jin,[a] Wei Wang,[a] Xitong Liu,[a] Haixiao Xu,[a] Jing Zhang,*[a] and Wei Huang*[ac]

[a] Key Laboratory for Organic Electronics and Information Displays & Jiangsu Key Laboratory for Biosensors, Institute of Advanced Materials (IAM), Jiangsu National Synergetic Innovation Center for Advanced Materials, Nanjing University of Posts & Telecommunications, 9 Wenyuan Road, Nanjing 210023, China
E-mail: iamjingzhang@njupt.edu.cn

[b] Department of Physics, Southern University of Science and Technology, Shenzhen 518055, China

[c] Shaanxi Institute of Flexible Electronics (SIFE), Northwestern Polytechnical University (NPU), 127 West Youyi Road, Xi'an 710072, Shaanxi, China E-mail: wei-huang@njtech.edu.cn

Contents

Experimental Procedures..........S-3

Fig. S1 Preparation of the TMTES-P/DTTCNQ single crystal S-7

Fig. S2 The intermolecular distance of the cocrystal P1 structure S-7

Fig. S3 Short-contact interactions in cocrystal P1..........S-8

Fig. S4 The intermolecular distance of the cocrystal P2 structure S-8

Fig. S5 Short-contact interactions in cocrystal P2..........S-9

Fig. S6 Infrared absorption spectra of pristine DTTCNQ and TMTES-P/DTTCNQ cocrystals obtained from chlorobenzene and toluene solvents..........S-9

Fig. S7 Solid-state UV-vis-NIR absorption spectra of the two cocrystals S-10

Fig. S8 Electron spin resonance (ESR) spectra of cocrystals P1 and P2 ....S-10

Fig. S9 Atomic force microscopy (AFM) images (10 × 10 μm) of P1 and P2 micro/nanoribbons S-11

Fig.S10 XRD images of microcrystals grew on SiO_2/Si substrates of at different annealing temperatures. S-11

Fig. S11 TEM image of the cocrystal P2 and its corresponding SAED pattern S-12

Table S1 Crystal data and structure refinement for cocrystal P1, P2, TMTES-P and DTTCNQ S-13

Supporting information references S-14

Experimental Procedures

Materials

1,4,8,11-tetramethyl-6,13-triethylsilylethynyl pentacene (TMTES-P) and 4,8-bis(dicyanomethylene)-4,8-dihydrobenzo[1,2-b:4,5-b']-dithiophene (DTTCNQ) were purchased from Ossila and Luminescence Technology Company, respectively. Chlorobenzene (C_6H_5Cl, HPLC) and toluene (C_7H_8, HPLC) were purchased from Sigma-Aldrich. All the materials were used directly without further purification.

Single Crystal Growth and Structural Analysis

For the growth of single crystal, the mixture of TMTES-P (1.2 mg) and DTTCNQ (0.6 mg) with molar ratio of 1:1 were dissolved in chlorobenzene (1 ml) and toluene (1 ml), respectively, and placed in reagent bottles. Then the prepared solutions were heated up to 110°C or 120°C lasted ~2 h to ensure complete dissolution. After that the as-prepared solution was put into a petri dish with approximately tilted 20°~30°. As the solvent slowly evaporates, dark black needle-like cocrystals can be observed at the bottom of the petri dish for approximately 3~4 days. Then cleaned with alcohol and dried in air. The single crystal structures of cocrystal P1 and P2 were obtained using a Bruker Smart-1000-CCD diffractometer, and using graphite-monochromated Mo Kα radiation (λ = 0.71073 Å). Exposure times of 10 s for all cocrystals were applied. The X-ray crystallographic data were collected at 277 K for cocrystal P1, 300 K for cocrystal P2. The structure was resolved by the direct method and refined by the full-matrix least-squares method on F^2 using the SHELXL-97 program, and analyzed using Mercury and Diamond. For all structures a symmetry-related (multi-scan) absorption correction was applied. The crystallographic data of

the crystal structure involved in this work has been uploaded to the Cambridge Crystallography Data Center. CCDC number: 1918869 (cocrystal P1), 1918870 (cocrystal P2).

Growth of the Self-Assembled Micro/nanocrystals and Device Fabrication

The organic field-effect transistors (OFETs) were constructed by using the top-contact/bottom-gate configuration. Here, highly *n*-doped Si wafers were employed as the gate electrode, with 500 nm thermally oxidized SiO_2 as the gate dielectric (the capacitance is 7.5 $nF \cdot cm^{-2}$). The silicon wafers were washed in turns with deionized water, hot piranha solution (H_2SO_4:H_2O_2=2:1), deionized water and isopropyl alcohol, then dried by a N_2 gun. After that, the surface of the wafer was modified with *n*-octadecyltrichlorosilane (OTS) self-assembled monolayer by thermally evaporating, and then the OTS modified wafers were washed with *n*-hexane, chloroform and isopropyl alcohol, dried under ambient N_2 before use. Cocrystal P1 and P2 were directly grown on the substrate by solution drop-casting method. A drop of chlorobenzene (0.9 mg/ml, molar ratio 1:1) or toluene (0.225 mg/ml, molar ratio 1:1) solution containing TMTES-P and DTTCNQ was poured over the bare SiO_2/Si and OTS-treated SiO_2/Si substrate in a nitrogen-filled glove box, respectively. Then the solvent evaporated at room temperature to obtain P1 and P2 micro/nanoribbons. Gold was thermally evaporated onto cocrystals as the source and drain electrodes (50 nm thickness), using copper grid as the shadow mask. All measurements were performed at room temperature in air condition. The morphology of micro/nanocrystals were observed by using an optical microscope (BX53, Olympus).

Measurements

The UV-vis-NIR absorption spectra were recorded by a LAMBDA 35 spectrometer. Infrared (IR) spectra of the cocrystals were recorded with a PE-Spectrum Two spectrometer, the single crystals

were mixed with KBr, completely grounded and then pressed into the slice for IR measurements. Powder X-ray diffraction (PXRD) was performed on a D/max2500 with Cu Kα source (κ = 1.5418 Å), the data were collected in the 2ϑ range 5-30° at room temperature. Elemental analyses were measured on a Vario MICRO elemental analyzer. ESR experiments were conducted on a Bruker a300 spectrometer (Bruker Co. Germany). The electrical measurements of OFETs were characterized using a Keitheley 4200 SCS semiconductor parameter analyzer and a Micromanipulator probe station in the ambient environment at room temperature. The charge-carrier mobility of the transistors was calculated in the saturation regime from the slope of the square root of the source-drain current $\sqrt{I_D}$ versus the gate-source voltage (V_{GS}), using the equation (1):

$$I_D = \left(\frac{W}{2L}\right) C_i \mu \left(V_{GS} - V_T\right)^2 \qquad (1)$$

Where I_D is the source–drain current, C_i is the capacitance per unit area of the dielectric layer, V_T is the threshold voltage, W and L are the channel width and length, μ is the charge carrier mobility, V_{GS} is the gate-source voltage, respectively.

Theoretical calculation

The calculation of transfer integral is based on neutral molecular orbitals.[1] Under several assumptions, the transfer integral is approximated by the coupling strength between two orbitals (HOMO and HOMO-1, or LUMO and LUMO-1), which can be expressed by Equation (2):

$$V_{ij} = < \psi_i | H | \psi_j > \qquad (2)$$

Where, V_{ij} is the coupling of orbital *i* and *j*, their corresponding wavefunctions are ψ_i and ψ_j. *H* is the Hamiltonian. Due to the electronic polarization of molecule, the effective transfer integral is corrected by Prof. Bredas,[2] which is the method we used here. The electronic couplings were calculated under the level of DFT/PW91PW91/6-31G (d), which was implemented in Gaussian 09.

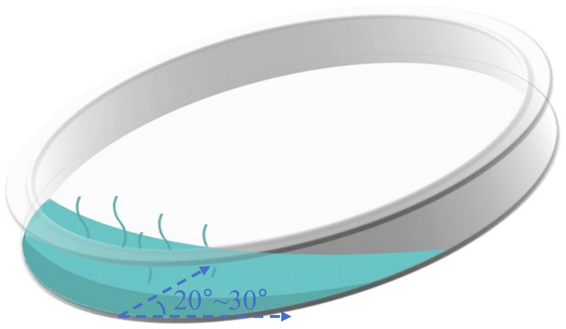

Fig. S1 Preparation of the TMTES-P/DTTCNQ single crystal with a 20°~30° orientation as the optimized condition for the solvent evaporation.

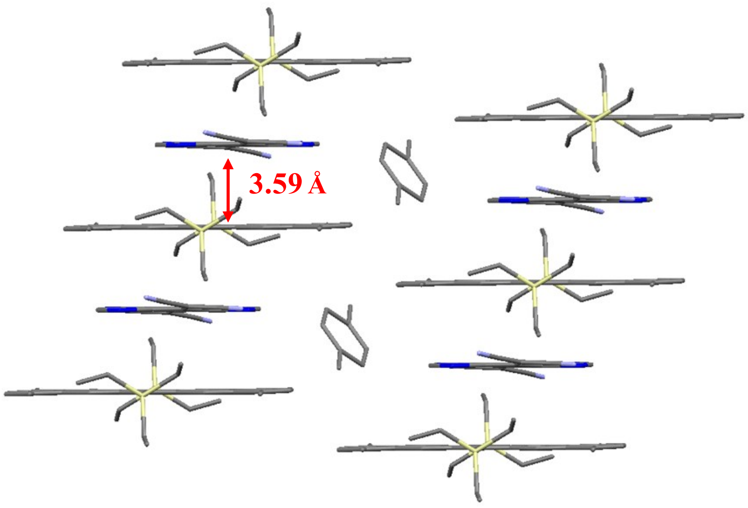

Fig. S2 The intermolecular distance of the cocrystal P1.

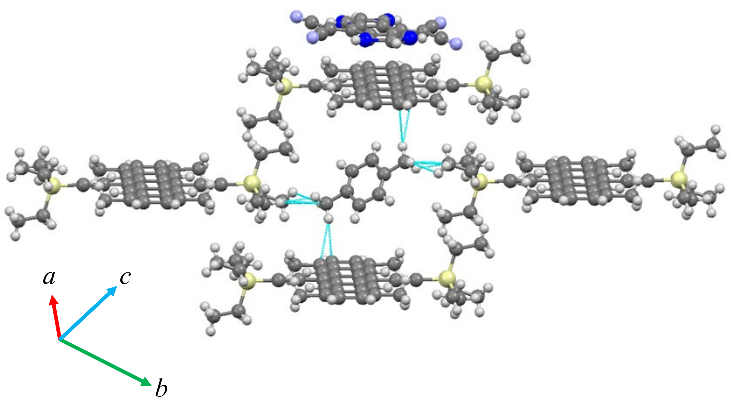

Fig. S3 Short-contact interactions in cocrystal P1.

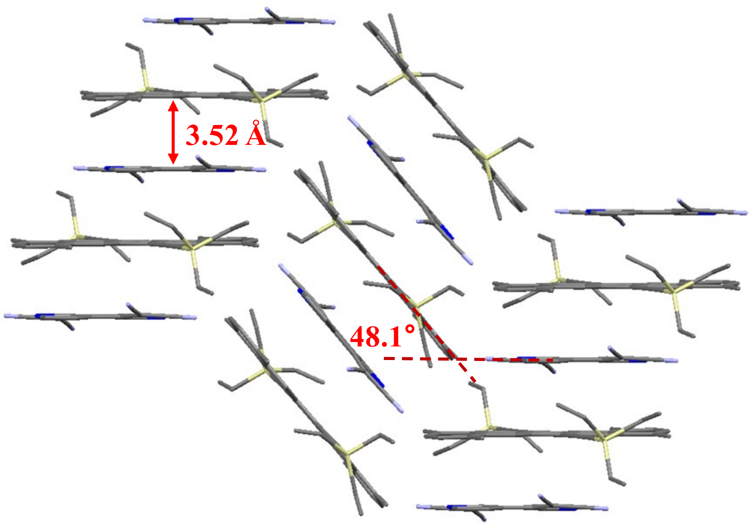

Fig. S4 The intermolecular distance of the cocrystal P2, the adjacent D-A stack columns adopted a 48.1° tilt angle.

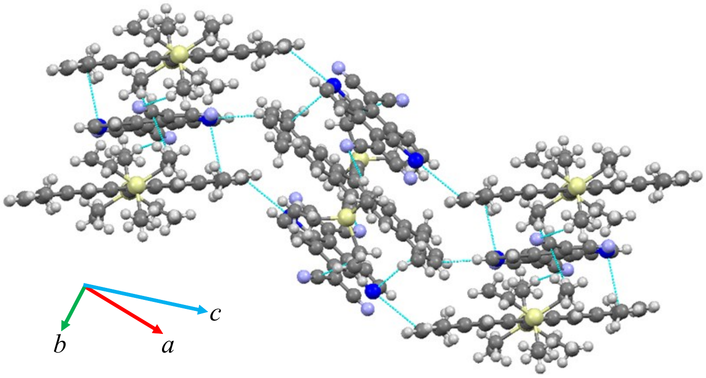

Fig. S5 Short-contact interactions in cocrystal P2.

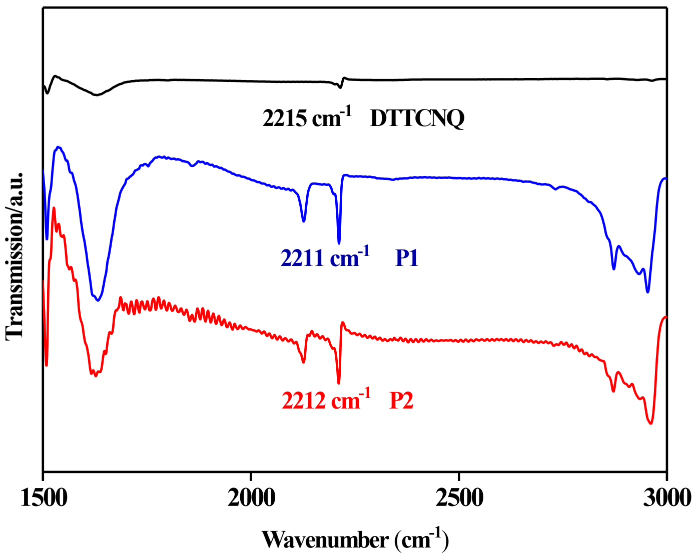

Fig. S6 Infrared absorption spectra of pristine DTTCNQ and TMTES-P/DTTCNQ cocrystal obtained from chlorobenzene and toluene solvents.

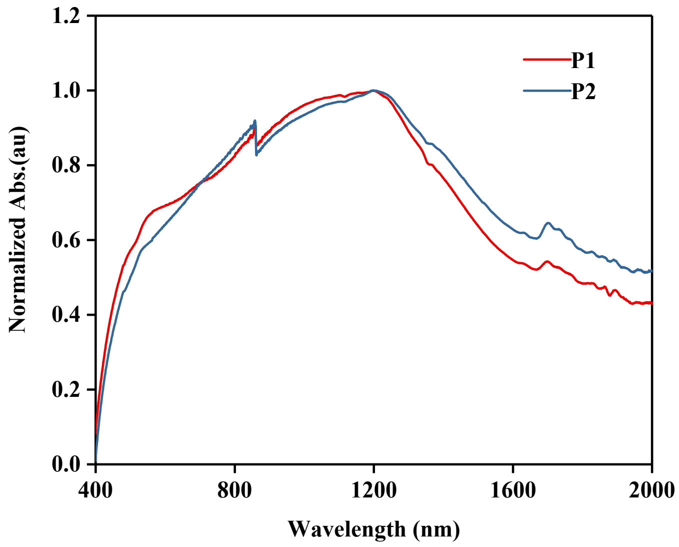

Fig. S7 Solid-state UV-vis-NIR absorption spectra of the two cocrystals.

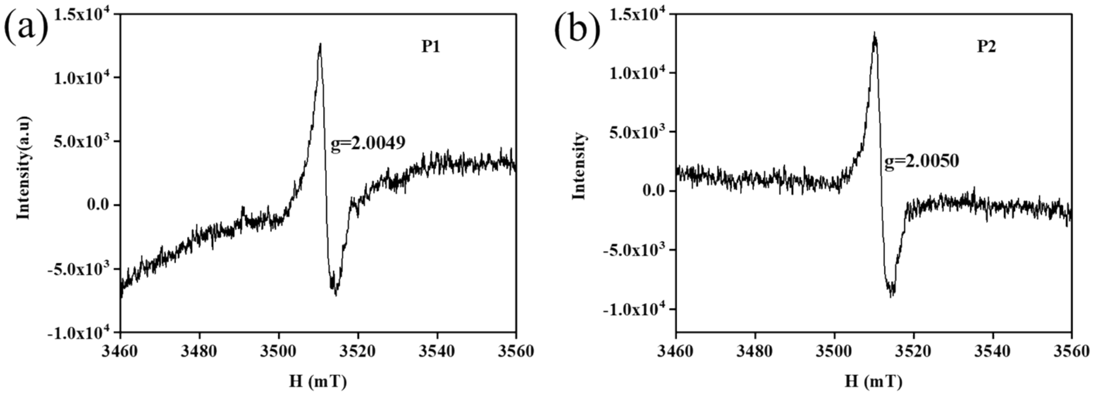

Fig. S8 Electron spin resonance (ESR) spectra of (a) cocrystals P1 and (b) P2.

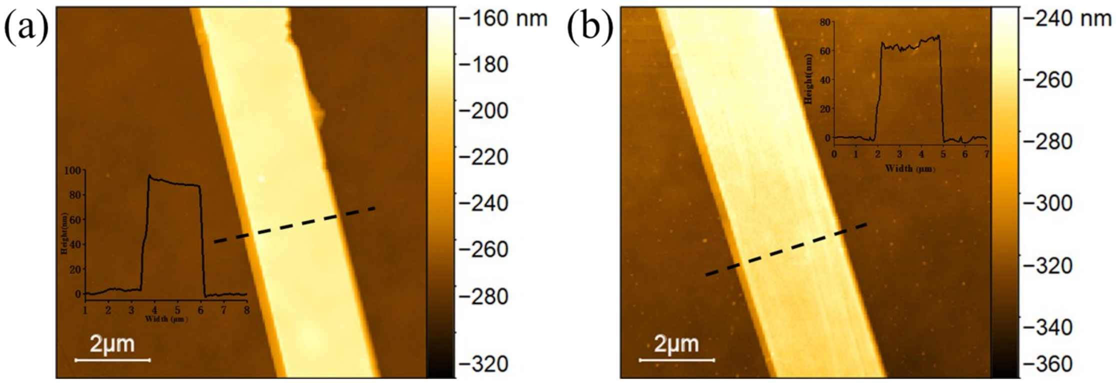

Fig. S9 Atomic force microscopy (AFM) images (10 × 10 μm) and related height plots of (a) P1 and (b) P2 micro/nanoribbons.

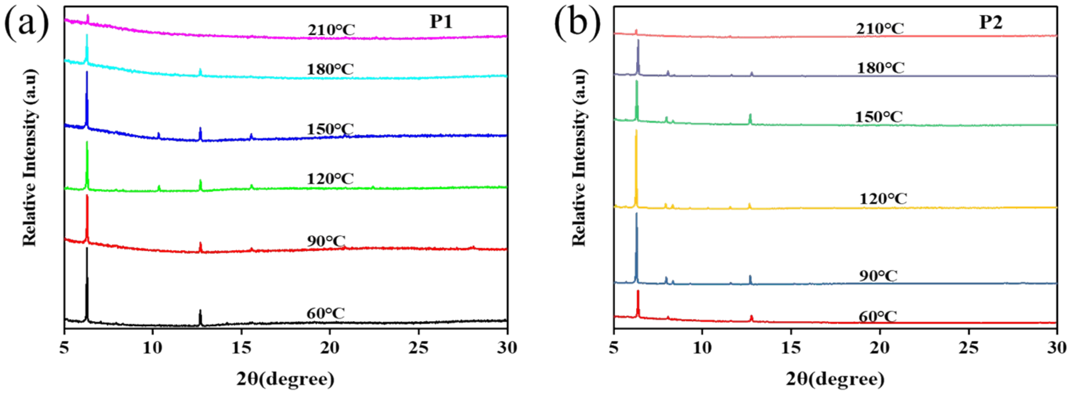

Fig.S10 XRD images of microcrystals grew on SiO_2/Si substrates of (a) P1and (b) P2 at different annealing temperatures.

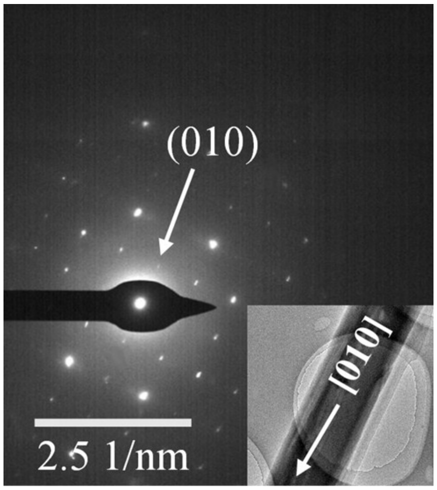

Fig. S11 TEM image of the cocrystal P2 and its corresponding SAED pattern.

Table S1 Crystal data and structure refinement for cocrystal P1, P2, TMTES-P and DTTCNQ data was also mentioned for comparison.

	P1	P2	TMTES-P[3]	DTTCNQ[4]
Empirical formula	$C_{66}H_{64}N_4S_2Si_2$	$C_{58}H_{54}N_4S_2Si_2$	$C_{42}H_{50}Si_2$	$C_{16}H_4N_4S_2$
Formula weight	1033.51	927.35	611.00	316.37
Temperature (K)	277(2)	300(2)		
Wavelength (Å)	0.71073	0.71073	0.71073	0.71069
Crystal system	Triclinic	monoclinic	Triclinic	Monoclinic
Space group	*P*-1	$P2_1/n$	P-1	$P2_1/n$
**Unit cell dimensions**				
a (Å)	7.727(4)	15.420(2)	7.3024(2)	16.588(3)
b (Å)	14.153(7)	7.710(11)	10.9762(3)	7.222(5)
c (Å)	15.295(7)	21.640(3)	12.1090(4)	11.353(3)
α (°)	109.638(12)	90	74.402(3)	90
β (°)	102.453(14)	104.049(4)	89.953(2)	89.88(2)
γ (°)	103.402(13)	90	73.746(2)	90
Volume ($Å^3$)	1451.7(12)	2495.8(6)	894.55(5)	1360.1(10)
Z	1	2	1	4
Absorption coefficient (mm^{-1})	0.176	0.197	0.127	0.374
Calculated density (g/cm^3)	1.182	1.234	1.134	1.545
F (000)	548.0	980.0	330	
Crystal size (mm^3)	0.500 × 0.300 × 0.200	0.500 × 0.300 × 0.200		
ϑ range for data collection (°)	2.610 to 24.999	1.94 to 25		13.6 to 16.75
**Limiting indices**				
	$9 \le h \le 9$<br>$-12 \le k \le 16$<br>$-18 \le l \le 18$	$-16 \le h \le 18$<br>$-9 \le k \le 9$<br>$-25 \le l \le 25$	$h_{max} = 8$<br>$k_{max} = 13$<br>$l_{max} = 14$	$-21 \le h \le 21$<br>$0 \le k \le 9$<br>$0 \le l \le 14$
Reflections collected	9307	13336		
Independent reflections	4999 [R(int) = 0.0239]	4216 [R(int) = 0.0532]		
Absorption correction	Semi-empirical from equivalents			
Refinement method	Full-matrix least-squares on F^2			
Data / restraints / parameters	4999/332/340	4216/362/342		
Goodness-of-fit on F^2	1.086	1.103		
Final R indices [I > 2sigma(I)]	$R_1 = 0.0997$<br>$wR_2 = 0.2893$	$R_1 = 0.1044$<br>$wR_2 = 0.2377$		
R indices (all data)	$R_1 = 0.1269$<br>$wR_2 = 0.3151$	$R_1 = 0.1468$<br>$wR_2 = 0.2558$	0.0771<br>0.1991	0.065<br>0.063

Supporting information references

1 (a) W. Q. Deng and A. W. William, *J. Phys. Chem. B*, 2004, **108**, 8614; (b) Y. Zhou, W. Q. Deng and H. L. Zhang, *J Chem Phys*, 2016, **145**, 104108; (c) Y. Zhou, G. Long, A. Li, A. Gray-Weale, Y. Chen and T. Yan, *J. Mater. Chem. C*, 2018, **6**, 3276.

2 E. F. Valeev, V. Coropceanu, D. A. da Silva Filho, S. Salman and J. L. Brédas, *J. Am. Chem. Soc.*, 2006, **128**, 9882.

3 G. R. Llorente, M. B. Dufourg-Madec, D. J. Crouch, R. G. Pritchard, S. Ogier and S. G. Yeates, *Chem. Commun.*, 2009, 3059.

4 I. Fujiko, T. Nobuhiko, H. Masamichi, Y. Norimasa, Y. Masanori and K. Keiji, *Bull. Chem. Soc. Jpn.*, 1992, **65**, 2173.
